# Efficient electrocatalytic reduction of CO_2_ on an Ag catalyst in 1-ethyl-3-methylimidazolium ethylsulfate, with its co-catalytic role as a supporting electrolyte during the reduction in an acetonitrile medium

**DOI:** 10.3389/fchem.2025.1515903

**Published:** 2025-04-09

**Authors:** Sayyar Muhammad, Asad Ali

**Affiliations:** ^1^ Department of Chemistry, Islamia College Peshawar, Peshawar, Khyber-Pakhtunkhwa, Pakistan; ^2^ Energy engineering, Division of Energy Science, Luleå University of Technology, Luleå, Sweden

**Keywords:** cyclic voltammetry, electrocatalysis, ionic liquids, co-catalyst, CO_2_ mitigation

## Abstract

CO_2_ electrochemical reduction reactions (CO_2_ERR) has shown great promise in reducing greenhouse gas emissions while also producing useful chemicals. In this contribution, we describe the CO_2_ERR at different catalysts using 1-ethyl-3-methylimidazolium ethyl sulfate [emim][EtSO_4_] ionic liquid (IL) as a solvent and as a supporting electrolyte. CO_2_ERR occurs at Ag and Cu catalysts at a lower overpotential than that at Au, Pt, and boron-doped diamond (BDD) catalysts. In addition, we report that ILs play a better co-catalytic role when used as a supporting electrolyte during CO_2_ERR in an acetonitrile (AcN) medium than the conventional supporting electrolyte, tetrabutylammonium hexafluorophosphate [TBA][PF_6_] in AcN. Furthermore, it is found that imidazolium-based cations ([emim]^+^) play a significant co-catalytic role during the reduction compared to [TBA]^+^ and pyrrolidinium [empyrr]^+^ cations, while anions of the ILs play no such role. The formation of CO from the CO_2_ERR was detected using cyclic voltammetry at an Ag catalyst both in [emim][EtSO_4_] as well as in an AcN solvent containing [emim][EtSO_4_] as a supporting electrolyte. The product of the CO_2_ reduction in this IL medium at the Ag catalyst is CO, which can be converted to synthetic liquid fuels by coupling the process with the Fischer–Tropsch process or through the conversion of CO_2_ into fuels based on green hydrogen by the Sabatier process, that is, methanation of CO_2_ on industrial scale, in the future.

## Graphical Abstract

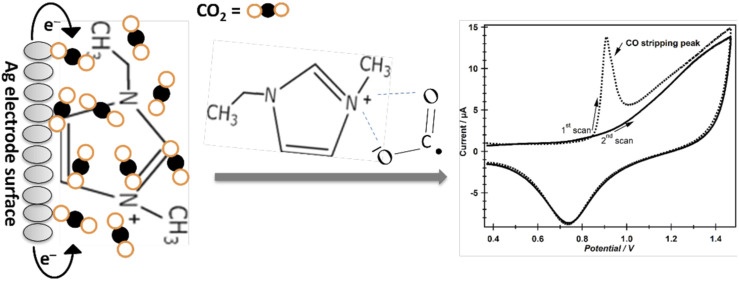

## 1 Introduction

Escalating emissions of carbon dioxide, CO_2_, have led to increasing efforts to combat its release. In addition to cutting emissions, we also need to take CO_2_ out of the atmosphere to stop the worst effects of climate change. We can lessen the amount of CO_2_ in the atmosphere and slow the rate at which global temperatures rise by converting atmospheric CO_2_ into other carbon-based fuel molecules by capturing and storing CO_2_ emissions. Lowering CO_2_ emissions protects biodiversity and lowers the likelihood of extreme weather occurrences by lessening the effects of climate change on ecosystems, sea levels, and weather patterns. CO_2_ mitigation and sequestration can assist in lowering emissions from current fossil fuel power plants and industrial processes as we switch from fossil fuels to renewable energy sources, paving the way for a cleaner energy future. Large volumes of CO_2_ are produced by several industrial processes, such as the fabrication of steel and cement. It may be possible to lower overall emissions from industries that are challenging to fully decarbonize by capturing CO_2_ from these processes and converting it to other useful molecules (Wu et al., 2022; Li et al., 2022; Guo et al., 2023). Overall, CO_2_ capture is a key tool in the broader strategy to address climate change and move towards a more sustainable future.

A range of chemical (Messou et al., 2021), biochemical (Rosen et al., 2012), photochemical (Dong et al., 2018; Lin et al., 2018), and electrochemical (Ávila-Bolívar et al., 2019; Faggion et al., 2019; Francke et al., 2018; Hiragond et al., 2020; Sánchez et al., 2019) methods have been used to fix CO_2_ into useful fuels and industrial chemicals. Electroreduction of CO_2_ is a significant and promising method for addressing climate change and converting greenhouse gases into valuable products, helping to mitigate its concentration in the atmosphere, recycling it into chemicals and fuels, including carbon monoxide (CO), methane (CH_4_), ethylene (C_2_H_4_), and alcohols like methanol, and ethanol, effectively turning a waste product into valuable resources (Mitchell et al., 2021; Resasco et al., 2018; Yuan et al., 2018). Electroreduction can be powered by renewable energy sources, such as solar or wind power. By turning extra power into chemical fuels, this improves process sustainability and facilitates the integration of renewable energy sources into the grid. The process can lower its dependency on fossil fuels and increase overall energy efficiency by utilizing excess renewable energy (Ikuerowo et al., 2024). In conclusion, the electroreduction of CO_2_ has two advantages: it reduces atmospheric CO_2_ to help slow global warming, and it produces useful products from what would otherwise be a waste product. Technology could become increasingly important in climate plans and sustainable energy systems as it develops.

Many commodity chemicals that may be used directly as fuel or as a fuel precursor are produced by the electroreduction of CO_2_ at metal electrodes in both aqueous and non-aqueous media. The selectivity of the electrochemical reduction of CO_2_ depends on the type of metal electrodes, the type of media (aqueous and non-aqueous), and the potential applied (Nakata et al., 2014; Sun et al., 2014; Yang et al., 2013). The commercialization of this technology is hampered by many issues, including the electrode’s instability, the desired product’s low Faradic efficiencies, and the high overpotential seen at various electrocatalysts and aqueous and non-aqueous electrolytes due to the stability of the CO_2_ molecule. In non-aqueous solvents such as dimethylformamide (DMF), the kinetically inert, stable, and linear-shaped CO_2_ is electrochemically reduced at various electrodes to a bent-shaped intermediated radical anion CO_2_^·−^ (CO_2_ + e^−^→ CO_2_^·−^) at a high overpotential (E^o^ of −1.97 V) (Benson et al., 2013; Marcandalli et al., 2021). This CO_2_^·−^ formation is a main hurdle in CO_2_ electrocatalysis. Any medium that can help convert this intermediated radical anion at low overpotential to useful fuel products is desirable.

Several efforts have been made to overcome the issue of high overpotential during CO_2_ERR, such as the development of electrocatalysts and searching for a new electrocatalytic medium (Usman et al., 2021; Mena et al., 2021). A significant amount of interest in the use of aprotic media such as ionic liquids (ILs) for the CO_2_ERR has arisen (Mitchell et al., 2021; Resasco et al., 2018; Yuan et al., 2018). Liquids that are completely composed of ions and that are liquid below 373 K are officially referred to as ILs. These liquids are regarded as a new and growing class of liquids that differ from traditional organic solvents and aqueous media. They are created by the weak electrostatic interaction of larger organic cations and anions of inorganic and/or organic nature. Aprotic ionic liquids (APILs) and protic ionic liquids (PILs) are the two primary categories of ILs, which are distinguished by whether they include any mobile conducting proton. While the latter are created by an acid–base reaction in which a proton is transferred from a proton donor acid to a proton accepter base and contain a labile conducting proton, the former are created by irreversible alkylation of a heteroatom and lack any conducting proton in their structure. They are being tested as potential solvents and electrolytes for a variety of electrochemical applications, such as batteries, supercapacitors, potential use as a proton conductor in intermediate temperature fuel cells, and CO_2_ capture, due to their important physiochemical properties, including their intrinsic conductivity, wide electrochemical windows (EWs), low vapor pressure, elevated thermal stability, and minimum fire-retardant ability (Muhammad et al., 2024; Muhammad et al., 2020; Muhammad et al., 2023).

Room temperature APILs are regarded as a promising electrolytic medium for electrochemical CO_2_ reduction because of their special characteristics. Imidazolium-based ionic liquids have attracted interest among room temperature APILs because of their superior electrochemical capabilities. Choi et al. (2016) showed that the overpotential for CO_2_ reduction may be considerably decreased by adding an ionic liquid, [Bmim][BF_4_]. In their investigation into the promotion of CO_2_ electroreduction, Zhang et al. (2024) used [Bmim][BF_4_], [Bmmim][BF_4_], [Bmim][PF_6_], [Bmmim][PF_6_], and [Bmim][NTf_2_]. They discovered that these Imim-ILs have a Faradic efficiency of CO of almost 95% and a high CO selectivity in CO_2_RR. These findings imply that Imim-ILs have a stimulating effect on CO_2_ electrochemical reduction, and in recent years, determining how they improve CO_2_ electrochemical reduction has become a major area of study (Gomes et al., 2024). Because of their great solubility in comparison to aqueous solutions and traditional organic solvents, as well as their high current density, product selectivity, and conversion efficiency, APIL electrolytes are generally suggested as a preferred medium for the electroreduction of CO_2_ (Zoski et al., 2021).

In neat APIL electrolytes, high viscosity can significantly affect mass transfer during a reaction. To enhance mass transfer in the system, it is common to mix the ionic liquid with a solvent to form a mixed electrolyte. AcN has emerged as a common solvent for CO_2_ERR due to its low viscosity and high CO_2_ solubility. Sun et al. (2014) highlighted the role of room temperature ILs as a supporting electrolyte during electroreduction of CO_2_ at Pb in acetonitrile. They found that the CO_2_ reduction overpotential in an acetonitrile solution decreases by using an ionic liquid as a supporting electrolyte compared to conventional tetraethylammonium perchlorate due to the catalytic role played by ionic liquids in changing the reduction pathway.

**SCHEME 1:**
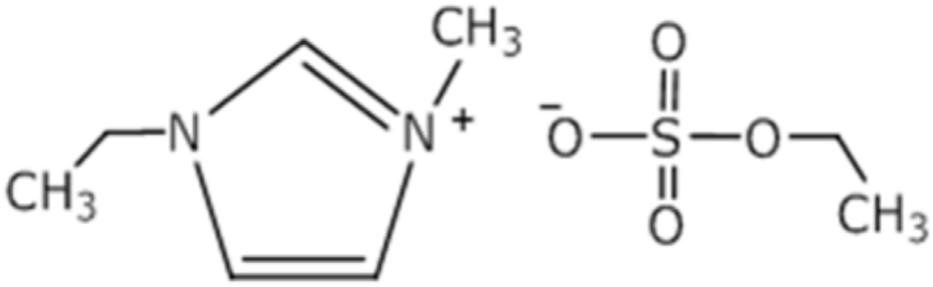
Chemical structure of [emim][EtSO_4_].

In this contribution, we report the electrochemical reduction of CO_2_ at Ag, Cu, Au, BDD, and Pt working electrodes using neat [emim][EtSO_4_] (Scheme 1) as the electrolyte. The results are compared, and a better electrocatalyst that undergoes CO_2_EER at a lower overpotential is identified. In many imidazolium-based ILs, such as [emim][Tf_2_N], [emim][Br] and [empyrr][EtSO_4_] (Resasco et al., 2018; Yuan et al., 2018), [Bmim][BF_4_] (Choi et al., 2016), [Bmim][BF_4_], [Bmmim][BF_4_], [Bmim][PF_6_], [Bmmim][PF_6_], and [Bmim][NTf_2_] (Zhang et al., 2024), the CO_2_ERR is reported. No such study has been carried out in [emim][EtSO_4_], which is why this IL is selected for this study. Different ILs were used as supporting electrolytes, and CO_2_ERR was investigated using acetonitrile as a non-aqueous medium. The results are compared with the CO_2_ERR using conventional [TBA][PF_6_] as the supporting electrolyte, and the role of the IL cation and anions during the electroreduction process is also elucidated.

## 2 Materials and methods

### 2.1 Reagents and apparatus

[emim][EtSO_4_] was acquired from Merck (≥95%). [emim][Br] was acquired from Across Organics (98%). [emim][Tf_2_N] and [empyrr][EtSO_4_] were kindly donated by Prof. Peter Licence’s ionic liquid research group of the School of Chemistry, University of Nottingham. Other chemicals used were AgNO_3_ (Fischer Scientific, analytical reagent grade), ferrocene (Alfa-Aesar, 99.0%), acetonitrile (BDH VWR Prolab, 99.9%), HClO_4_ (Fischer Chemicals, Fischer Scientific, United Kingdom, 60%), and tetrabutylammonium hexafluorophosphate [TBA][PF_6_] (Sigma-Aldrich, 98%). CO_2_ (99.5%), N_2_ (99.9999%), and Ar (99.995%) were acquired from BOC, Ltd. (Nottingham, United Kingdom). The compounds were utilized in their original form. A model 760C potentiostat from CH Instruments (Austin, TX, United States) was used to conduct the electrochemical experiments.

### 2.2 Instrument and electrodes for electrochemical measurements

We performed cyclic voltammetry (CV), linear sweep voltammetry (LSV), and chronoamperometry (CA) experiments in triplicate for data reproducibility check, using a three-necked glass cell and a conventional three-electrode configuration. The CV, LSV, and CA experimental data were found to be reproducible. The working electrodes were BDD, Au, Cu, and Ag disks of geometrical surface area 7.07 × 10^−2^ cm^2^, 3.14 × 10^−2^ cm^2^, 3.14 × 10^−2^ cm^2^, 3.04 × 10^−2^ cm^2^, and 2.51 × 10^−2^ cm^2^, respectively. The counter electrode used was a Pt flag (0.5 cm × 0.4 cm). For the Au electrode, ECSA was determined from the CV obtained in 0.1 M aqueous HClO_4_ solution by the electronic charge of gold oxides (Ma et al., 2014) and was 9.5 × 10^−2^ cm^2^. The ECSA of the Ag and Cu electrodes were estimated to be 4.78 × 10^−2^ cm^2^ and 4.11 × 10^−2^ cm^2^, respectively, from the charge passed during the under-potential deposition of lead onto each electrode from 5 mM Pb (NO_3_)_2_ solution in 10 mM KCl/10 mM HNO_3_ (Supplementary Figures S1, S2 in the supporting information) (Salehi-Khojin et al., 2013; Lu et al., 2012). The charge under the Pb_UPD_ stripping peak at a Pb^2+^ concentration of 5 mM or above corresponds to 600 μC cm^−2^ Ag. The same integrated charge density under the Pb_UPD_ region was used to estimate the ECSA of the Cu electrode from a CV obtained at a Cu electrode (Supplementary Figure S2 in the supporting information) in 5 mM Pb (NO_3_)_2_ solution in 10 mM KCl plus 10 mM HNO_3_ (Rosen et al., 2012). The currents in each measurement were normalized to the ECSA for calibration of catalytic activity of the electrodes for the CO_2_ reduction in the room temperature ILs. In the case of the BDD electrode, the geometric surface area of the electrode was used to calibrate the currents for CO_2_ reduction in the liquids. The reference electrode was a self-made Ag/Ag^+^ reference electrode, which was normalized *versus* Fc/Fc^+^, and the results are reported vs. Fc/Fc^+^.

### 2.3 Construction and calibration of Ag/Ag^+^ reference electrodes

Ag/Ag^+^ reference electrodes were constructed using a 7.5-cm-long glass tube, a 3.2 mm porous Vycor frit, and a heat-shrink Teflon tubing from Gamry Instruments, United States. The Vycor tip was placed inside the piece of a heat-shrink tube and held to the glass capillary by sliding the heat-shrink Teflon over the end of the glass tube. The Teflon was then gently warmed with a heat gun until the tubing tightened around the Vycor tip and the end of the reference electrode tube. The electrode was then filled with 10 mM AgTfO (Sigma-Aldrich ≥98.0% (Ag) in [emim][EtSO_4_], and an Ag wire connected to a Teflon top was immersed. In the study that involved AcN as a main electrolyte, Ag/Ag^+^ reference electrodes were made by the same procedure, but the Ag wire was immersed in 10 mM AgNO_3_ in either 0.1 M [TBA][PF_6_]/AcN or 0.1 M [emim][EtSO_4_]/AcN. Reference electrode stability is a common issue when using ionic liquids as electrolytes in electrochemistry (Zhao et al., 2008). Therefore, the Ag/Ag^+^ electrode (Ag/AgTfO in [emim][EtSO_4_]) was calibrated against the potential of the IUPAC-recommended ferrocene/ferrocenium (Fc/Fc^+^), redox couple (Ma and Kenis, 2013; Niu et al., 2015a). Supplementary Figure S3 in the supporting information shows CVs obtained in 5 mM ferrocene solution in [emim][EtSO_4_] at Pt and Au electrodes at 100 mV s^−1^. The reference electrodes containing 10 mM AgNO_3_ in 0.1 M [emim][EtSO_4_]/AcN and 0.1 M [TBA][PF_6_]/AcN were also calibrated *versus* ferrocene in 0.1 M [TBA][PF_6_]/AcN and 0.1 M [emim][EtSO_4_]/AcN, respectively (Supplementary Figure S4 in the supporting information). The formal potential, E^0^΄ (taken as the average of the Ferrocene (Fc) oxidation peak potential and cathodic ferrocenium (Fc^+^) reduction peak potential), was determined in N_2_-saturated solution vs. the Ag/Ag^+^ reference electrodes, and all potentials are reported *versus* this.

### 2.4 Electrochemical methods

The electrodes were polished before use using soft polishing pads and an aqueous suspension of 0.05 μm alumina from Buehler, Lake Bluff, Illinois (Buehler consumable, USA). The electrodes were cleaned, thoroughly rinsed with deionized water, subjected to 2 min of sonication in a very small amount of deionized water, and then thoroughly rinsed once more with deionized water before being dried in a stream of N_2_. The cell containing roughly 5 mL of [emim][EtSO_4_] was submerged with the working, counter, and reference electrodes. After the solution was bubbled for 30 min with either N_2_ or Ar to eliminate dissolved oxygen or for 60 min with CO_2_ (the ideal duration for CO_2_ saturation in the IL), cyclic voltammograms or LSVs were captured. To study the effect of temperature on CO_2_ERR in [emim][EtSO_4_], we did the celebration of the Ag/Ag^+^ reference electrode at room temperature and also at 50°C, 80°C, and 100°C. Before recording an LSV for CO_2_ERR at each temperature, CO_2_ gas was purged in the IL for 60 min.

### 2.5 Electrochemical analysis of the product of CO_2_ reduction

A 3-electrode system was adopted for the electrochemical analysis of the product formed by the reduction of CO_2_ in [emim][EtSO_4_], as described above. However, an additional 2 mm-diameter Pt electrode was used as the second working electrode to adsorb CO if any was formed from CO_2_ reduction at the Ag working electrode. The experiment was performed using an Ag/Ag^+^ reference electrode and a Pt flag counter electrode. Chronoamperometry measurements (current-time transient) were carried out by holding the Ag working electrode for 2,400 s at a −2.33 V vs. Fc/Fc^+^ redox couple in CO_2_-saturated [emim][EtSO_4_] and also in [emim][EtSO_4_]/AcN. A 2 mm-diameter freshly polished Pt disk electrode (activated in aqueous HClO_4_) was held at −0.4 V vs. Fc/Fc^+^ in the same solution for adsorption of the CO forming during the reduction reaction. After the experiment, the Pt electrode was removed from the CO_2_/IL system and rinsed with de-ionized water, then immersed in 0.1 M aqueous HClO_4_ in a three-necked cell to voltammetrically strip off any CO adsorbed on the electrode surface.

## 3 Results and discussion

### 3.1 CO_2_ERR in [emim][EtSO_4_]

**FIGURE 1:**
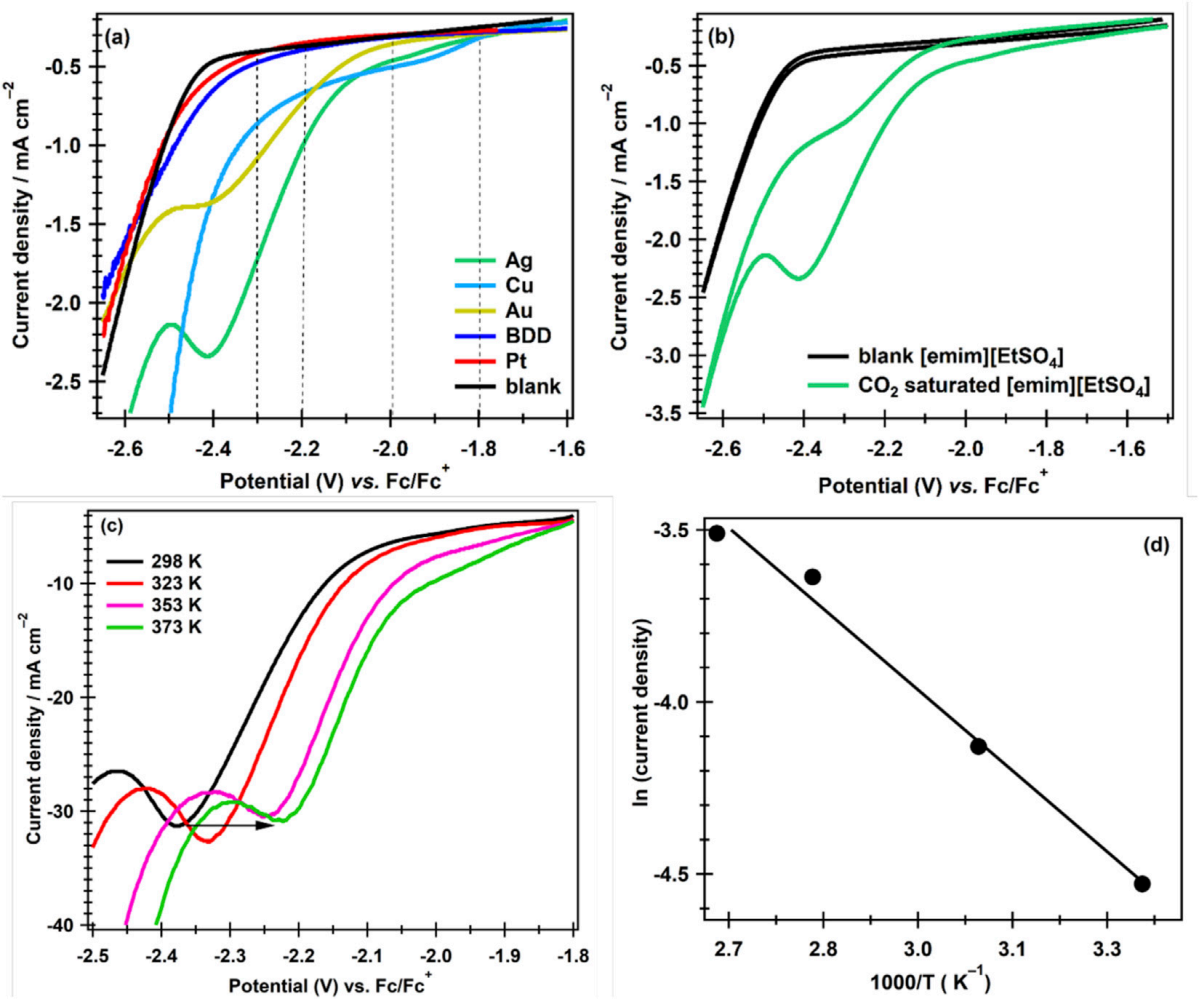
**(a)** LSV profiles for CO_2_ERR in [emim][EtSO_4_] saturated with CO_2_-for 60 min **(b)** CVs obtained at an Ag electrode in Ar-saturated (black CV) and CO_2_-saturated [emim][EtSO_4_] (green CV). **(c)** LSV profiles were recorded in CO_2_-saturated [emim][EtSO_4_] solution at 298 K, 323 K, 353 K, and 373 K on an Ag electrode at a scan rate of 100 mV s^−1^. **(d)** Shows the Arrhenius plot of CO_2_ERR in the IL in the temperature range of 298–373 K.

Figure 1a shows the cathodic sweep profiles obtained in CO_2_-saturated [emim][EtSO_4_] at Ag, Cu, Au, BDD, and Pt electrodes. The initial scan direction was from positive toward negative potentials. CVs were obtained at these catalysts (electrodes), and the initial half of the CV (LSV) is reported here. By comparing the CO_2_ electroreduction activity of these electrodes with that of the blank LSV (without CO_2_), it is evident that some reduction of CO_2_ is observed at each of these electrodes. However, by comparing the CO_2_ reduction LSVs obtained at the studied electrodes, it is observed that at both the Ag and Cu electrodes, the onset potential for the reduction of CO_2_ was −1.8 V compared with the onset potentials at Au (−2.0 V), BDD (−2.2 V), and Pt (−2.3 V), respectively. Thus, according to the current study, both Ag and Cu are better electrocatalysts for the electrocatalytic reduction of CO_2_ in [emim][EtSO_4_] medium, while at the Pt electrode, the electrocatalytic reduction of CO_2_ occurs at a very high overpotential (0.5 V) compared to the Ag and Cu electrocatalysts used for CO_2_ERR followed by BDD, at which the CO_2_ reduction occurs at a 0.4 V overpotential and the overpotential observed at the Au electrode compared to Pt and BDD is 0.2 V. Although CO_2_ERR occurs at the same onset potentials at both the Ag and Cu catalysts, a wide reductive wave with a higher current density is observed at the Ag than at the Cu catalyst. In addition, Ag is found to have known product selectivity that reduces CO_2_ mainly to CO (Salehi-Khojin et al., 2013; Niu et al., 2015b; Sampson et al., 2014; Huang et al., 2009). Therefore, Ag was mainly used as a catalyst for further investigation of the CO_2_ electroreduction in [emim][EtSO_4_] and other ILs.

Figure 1b shows cyclic voltammograms of CO_2_-saturated (green CV) and CO_2_-free, that is, Ar-saturated [emim][EtSO_4_] (black CV) recorded at an Ag electrode at 100 mV s^−1^ at room temperature. In the Ar-saturated CV, only the background charging current flowed between −1.5 V and −2.5 V. After −2.5 V, a sharp increase in the reduction current started, which is attributed to the cation, [emim]^+^, reduction of the IL. In the CO_2_-saturated liquid, a single irreversible broad reduction wave was observed at an onset of −1.8 V vs. Fc/Fc^+^, which peaked at approximately −2.4 V. The increase in the reduction current between −1.8 V and −2.5 V is attributed to an irreversible one-electron electroreduction of CO_2_ to CO_2_^·−^ in the IL (Rosen et al., 2012; Sánchez et al., 2019; Ma and Kenis, 2013). It is also evident from the figure that during the reverse sweep, a small reductive wave between −2.4 V and −2.3 V is seen, which is also due to the electroreduction of CO_2_ on Ag catalysts.

APILs are thermally stable and can also be used for high-temperature studies. Room temperature is frequently used to study the CO_2_ERR and practical electrolyzers; however, higher temperatures might be employed. Although it is well understood that the rates of reactions increase with an increase in temperature, fundamental knowledge of how temperature affects CO_2_ERR is currently lacking. To learn more about how temperature affects this reaction, we conducted temperature-dependent investigations on the CO_2_ERR using an Ag electrode. Figure 1c shows LSV profiles for CO_2_ERR in [emim][EtSO_4_] obtained at 298 K, 323 K, 353 K, and 373 K using polycrystalline an Ag disk electrode. Initially, CVs were recorded in the solution in a potential range from −1.8 V to −2.5 V vs. Fc/Fc^+^, and only the cathodic sweeps are shown in the figure. It is clear from the figure that at high temperatures, Ag shows better catalytic activity for CO_2_ reduction. The onset potential and the reduction peak potential shift toward the more positive potential. The overpotential for CO_2_ reduction in the ionic liquid decreased by ∼100 mV, and the onset reduction peak potential shifted from −2.37 V to −2.21 V when the temperature increased from 298 K to 373 K.

The Arrhenius equation ( $\ln K=lnA-\frac{E_{a}}{RT}$ ) can be used to determine the apparent activation energy for the CO_2_ERR on Ag based on the temperature-dependent experiments. In the Arrhenius equation, k is the reaction rate constant, A is the pre-exponential factor, Ea is the activation energy, R is the gas constant (8.3143 J. K^−1^. mol^−1^), and T is the absolute temperature. Figure 1d shows the Arrhenius equation ( $\left.\ln j=lnA-\frac{E_{a}}{RT}\right)$ plot of the natural logarithm of the current density (ln j in mA cm^−2^) *versus* 1/T (K^−1^) at a constant potential of −2.2 V. It can be seen from the figure that the current density increases with an increase in the temperature; that is, for a lower 1/T value, a smaller value of current density is observed, and for a higher 1/T, value the magnitude of the current density increased. The higher intercept value from the graph indicates a higher ln j value, as from the Arrhenius equation, ln j is directly proportional to ln A. A small value of the activation energy (E_a_), 13.04 J mol^−1^ determined from the slope of the plot, shows that the CO_2_ERR occurred at a fast rate at the Ag catalysts in the [emim][EtSO_4_]. Lv et al. (2020) reported the activation energies for the [TETAH][Lys]-ethanol-water and [TETAH][Lys]- water solutions capturing CO_2_ as 61.42 kJ/mol and 62.10 kJ/mol, respectively. Wu et al. (2016) reported the calculated activation energy of 41.89 kJ mol^−1^ for CO_2_ capture in 1-butyl-3-methylimidazolium glycinate aqueous solutions. Vos and Koper (2022) studied the effect of temperature on the cation-promoted electrochemical CO_2_ reduction on gold and reported 60 kJ mol^−1^ apparent activation energy with constant CO_2_ concentration. Thus, compared to the literature-reported activation energy values for CO_2_ reduction/capture in CO_2_-saturated ILs, the activation energy calculated in this study is smaller, showing that an Ag catalyst surface, or the cation [emim]+, or the synergistic approach of both, activate the CO_2_ERR to take place at lower overpotentials. The [emim]+ ---CO^∙−^, on the one hand, reduces the activation energy for the reaction. On the other hand, the formation of surface intermediates like formate (HCOO) and carboxyl (COOH) species on the surface of thermally activated Ag can result in the final product at a fast rate (Álvarez et al., 2017). The data fit statistically well, as the regression coefficient, R^2^, obtained from the Arrhenius plot is 0.9899, which is near the ideal fit R^2^ = 1. The analysis confirms that the rate of the CO_2_ERR increased as the temperature increased. At lower temperatures, ILs are more viscous. The viscosity of the IL decreases with increasing temperature, causing faster mass transport of CO_2_ toward the electrode surface (Zare et al., 2019).

### 3.2 Determination of the diffusion coefficient and concentration of CO_2_

**FIGURE 2:**
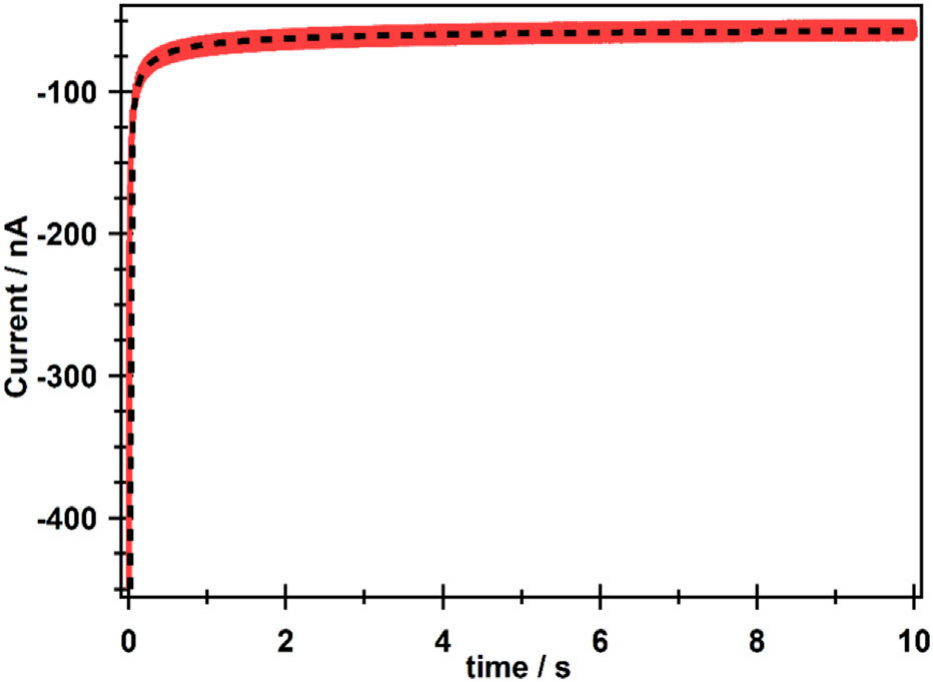
Experimental (red line) and theoretically fitted (black dotted line) chronoamperometric transients for the reduction of CO_2_ in [emim][EtSO_4_] at room temperature on a 15.6 μm-radius Au electrode.

The diffusion coefficient (D) and concentration (*C*) of CO_2_ in [emim][EtSO_4_] were experimentally obtained by a potential-step chronoamperometry. A chronoamperometric transient was recorded using an Au microelectrode (nominal radius 12.5 µm) and is shown in Figure 2 (red line). The potential was stepped from −1.8 V vs. Ag/Ag^+^ (−2.05 V vs. Fc/Fc^+^) to −2.3 V (−2.55 V vs. Fc/Fc^+^) for 10 s. The black dotted line in Figure 2 shows the theoretical fitted data using the Shoup–Szabo equation shown as Equation 1, which allows determining both D and C of CO_2_ simultaneously (Bard, 2010; Spendelow et al., 2004).

$$i=-4\;n\;F\;D\;C\;rf\tau \left(\tau \right),$$ (1)

where n is the number of electrons involved in the redox process, which is taken as 1 for the reduction of CO_2_ to CO_2_^·−^, and r is the radius of the Au microelectrode, which was determined using steady-state current. i_ss_ is used for oxidation of ferrocene at the microelectrode and is determined using Equation 2 (Supplementary Figure S5 in the supporting information) (Bard, 2010).

$$i_{ss}=4\;n\;F\;C\;D\;r,$$ (2)

where i_ss_ was 7.97 × 10^−9^ A, and n is the number of electrons involved in the redox process, which is 1 for the Fc/Fc^+^ redox couple. The concentration, c, of the ferrocene solution was 2 × 10^−6^ mol cm^−3^. The diffusion coefficient of the Fc/Fc^+^ redox couple was 6.7 × 10^−6^ cm^2^ s^−1^ (Bard, 2010; Bond et al., 2010). The actual radius of the Au microelectrode calculated through this experiment was 15.6 µm.

The function f ( τ ) and the parameter, τ in the Shoup–Szabo equation are given by Equations 3, 4, respectively.

$$f\left(\tau \right)=0.7854+0.8863\tau^{-1/2}+0.2146\exp\left(-0.7823\tau^{-1/2}\right).$$ (3)

$$\tau =4\;Dt/r^{2}$$ (4)

The D and *C* values obtained from theoretical fitted data were 4.78 × 10^−10^ m^2^ s^−1^ (or 4.78 × 10^−6^ cm^2^ s^−1^) and 1.83 × 10^−5^ mol cm^−3^ (0.0183 mol L^−1^), respectively.

The diffusion coefficient of CO_2_ in [emim][EtSO_4_] (η = 108 cP) is comparable to the diffusion coefficient of O_2_ in [bmim][BF_4_] (η = 92 cP), which was 1.79 × 10^−10^ m^2^s^−1^ measured at 298 K using a 10-µm-diameter Au electrode (Martindale and Compton, 2012). However, the D value for CO_2_ in [emim][EtSO_4_] was higher by two orders of magnitude than that observed in [bmim][Ac] (Wang et al., 2020), which was 2.65 × 10^−12^ m^2^s^−1^. This could be due to the low viscosity of [emim][EtSO_4_], in which CO_2_ molecules can diffuse faster than [bmim][Ac] (η = 140 cP). However, viscosity could not be the only reason for the high diffusion of CO_2_ in [emim][EtSO_4_]. The arrangement of atoms or alkyl groups around the central atom in the cation and anion may also play a role. For example, [bmim][BF_4_] has viscosity η = 112 cP, which is very close to that of [emim][EtSO_4_], 108 cP (Samjeské et al., 2009), and the D value for CO_2_ in the latter case is higher by an order of magnitude than the former (7.3 × 10^−11^ m^2^s^−1^) (Wang et al., 2020).

### 3.3 [emim][EtSO_4_] as supporting electrolyte during CO_2_ ERR in AcN

**FIGURE 3:**
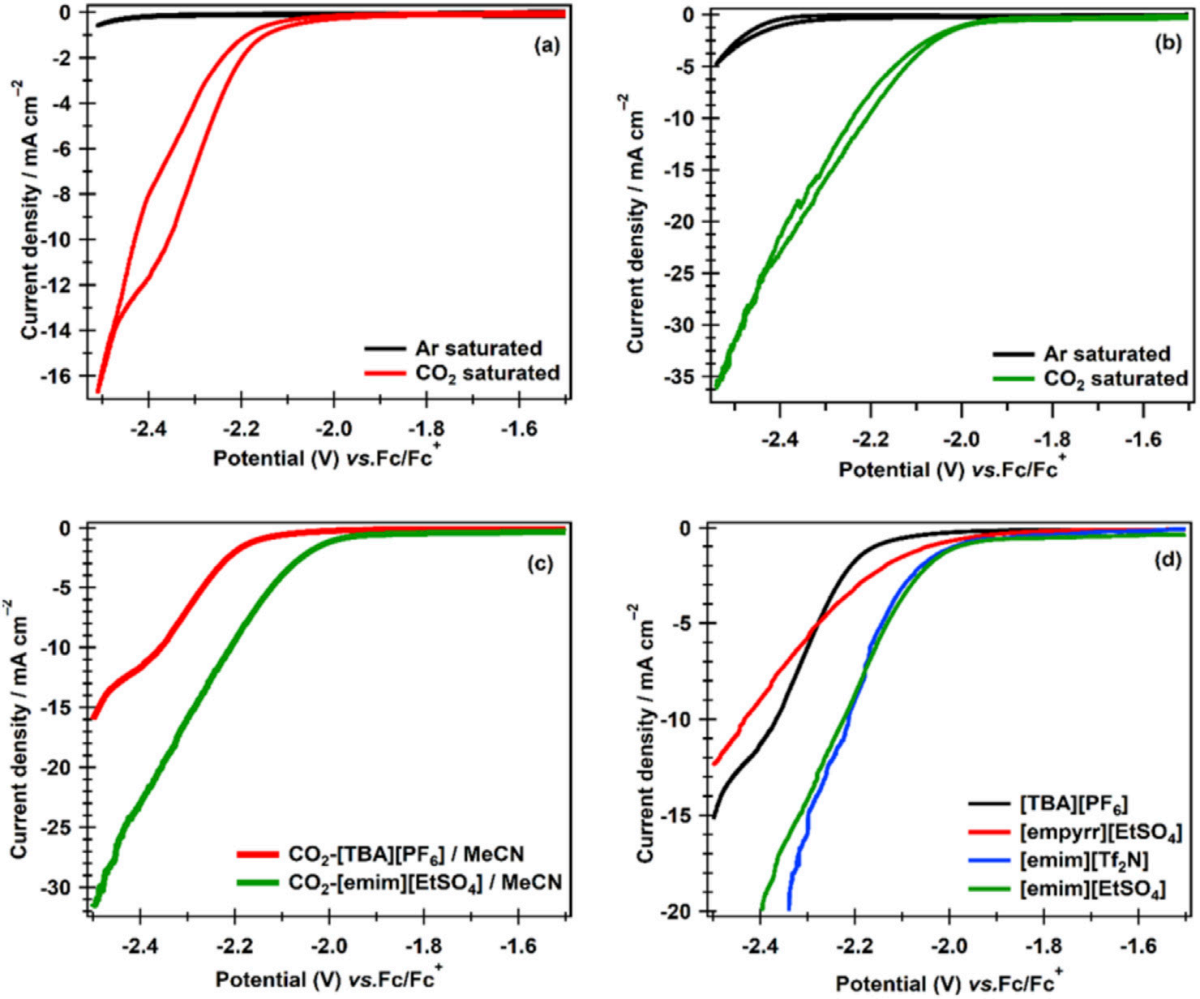
CVs recorded at an Ag catalyst at a scan rate of 100 mV s^−1^ in a potential range from −1.7 V to −2.5 V in **(a)** 0.1 M [TBA][PF_6_]/AcN solution, and **(b)** 0.1 M [emim][EtSO_4_]/AcN solution after bubbling Ar for 30 min and then CO_2_ for 60 min into each solution. **(c)** Shows a comparison of the cathodic sweeps for CO_2_ ERR. **(d)** Shows LSV profiles obtained in a CO_2_-saturated solution of AcN containing 0.1 M of each [TBA][PF_6_], [empyrr][EtSO_4_], [emim][Tf_2_N], and [emim][EtSO_4_] as supporting electrolyte.

The effect of [emim][EtSO_4_] as a supporting electrolyte and/or co-catalyst was investigated during CO_2_ERR in the conventional organic solvent, AcN, and the results were compared with the CO_2_ERR carried out in AcN using the conventional supporting electrolyte, [TBA][PF_6_]. Figure 3 shows cyclic voltammograms measured in AcN containing (a) 0.1 M [TBA][PF_6_] and (b) 0.1 M [emim][EtSO_4_] at a 2 mm-diameter Ag electrode. Each of the solutions was purged with Ar for 30 min to remove dissolved oxygen, as oxygen can actively reduce and hinder the main electrochemical process, and CVs were recorded as shown by black lines in the Figures. Then, CO_2_ was bubbled for 60 min into the solutions, and CVs were measured at an Ag catalyst, as shown by the red and green lines in Figures 3a, b, respectively. In the blank (Ar saturated) [TBA][PF_6_]/AcN, only capacitive current flowed up to −2.48 V, after which a large reductive current started flowing that corresponds to the decomposition of AcN. In CO_2_-saturated [TBA][PF_6_]/AcN, the current response was different from that in blank solutions. A reduction current started flowing at an onset potential of −2.1 V until −2.48 V. This cathodic reduction wave, which was also observed during the reverse sweep, is attributed to the electrocatalytic CO_2_ reduction at the Ag catalyst in the solution.

Similarly, if we compare the CV obtained in the CO_2_-saturated [emim][EtSO_4_]/AcN solution (Figure 3b) with the blank CV (Ar-saturated), a sharp increase in the current density can be seen at an onset potential of −1.9 V. The reduction current kept increasing and can also be observed during the reverse sweep, which is attributed to the CO_2_ electroreduction. This shows the Ag electrode’s ability to reduce CO_2_ in AcN using both [TBA][PF_6_] as well as [emim][EtSO_4_] as supporting electrolytes. However, it is clear from Figure 3c that the reduction of CO_2_ occurs at ∼200 mV lower potential when using [emim][EtSO_4_] as a supporting electrolyte in AcN than when [TBA][PF_6_] is used as a supporting electrolyte. The high current density and early onset potential for CO_2_ electroreduction show that [emim][EtSO_4_] plays a co-catalytic role. This could be attributed to the fact that [emim]^+^ may stabilize the intermediate radical anion, CO_2_^·−^ during the intermediate step of the reduction of CO_2_, that is, the electron uptake process (CO_2_ + e− → CO_2_^·−^) and further help in its reduction (Niu et al., 2015b; Sampson et al., 2014).

As mentioned above, [emim][EtSO_4_] has some electrocatalytic effect toward CO_2_ERR when used alone as a non-aqueous medium or as a supporting electrolyte in AcN at an Ag catalyst. To verify whether cation or anion of the ILs plays a role during CO_2_ERR, experiments were performed using different ionic liquids as supporting electrolytes in AcN. Figure 3d displays linear sweep voltammograms for the reduction of CO_2_ in AcN containing 0.1 M of each of [TBA][PF_6_], [empyrr][EtSO_4_], [emim][Tf_2_N], and [emim][EtSO_4_] obtained at a scan rate of 100 mV s^−1^on an Ag catalyst. Generally, it is clear from the figure that compared to [TBA][PF_6_], the CO_2_ ERR occurs at a lower onset potential when ILs are used as supporting electrolytes in AcN. This confirms further that ILs play a co-catalytic role during the reduction process. Furthermore, it can be seen from the figure that a low overpotential is observed for the CO_2_ERR when [emim]^+^ cation-based ILs, that is, [emim][EtSO_4_] and [emim][Tf_2_N], were used as supporting electrolytes as in these ILs. The onset potential for the reduction is approximately the same (∼1.9 V), although the anions are different. However, while comparing the catalytic role of the different ILs toward CO_2_ERR, it can be seen that the overpotential for the reduction of CO_2_ in AcN solution containing 0.1 M [empyrr][EtSO_4_] as a supporting electrolyte is higher by 80 mV than that containing [emim][EtSO_4_] and [emim][Tf_2_N]. This shows that the imidazolium-based ILs, especially [emim]^+^, play some co-catalytic role during the reduction of CO_2_ at the Ag catalyst, also signifying the role of the cation of the ILs during CO_2_ electrocatalysis [51].

### 3.4 Electrochemical analysis of the product formed during CO_2_ reduction

**FIGURE 4:**
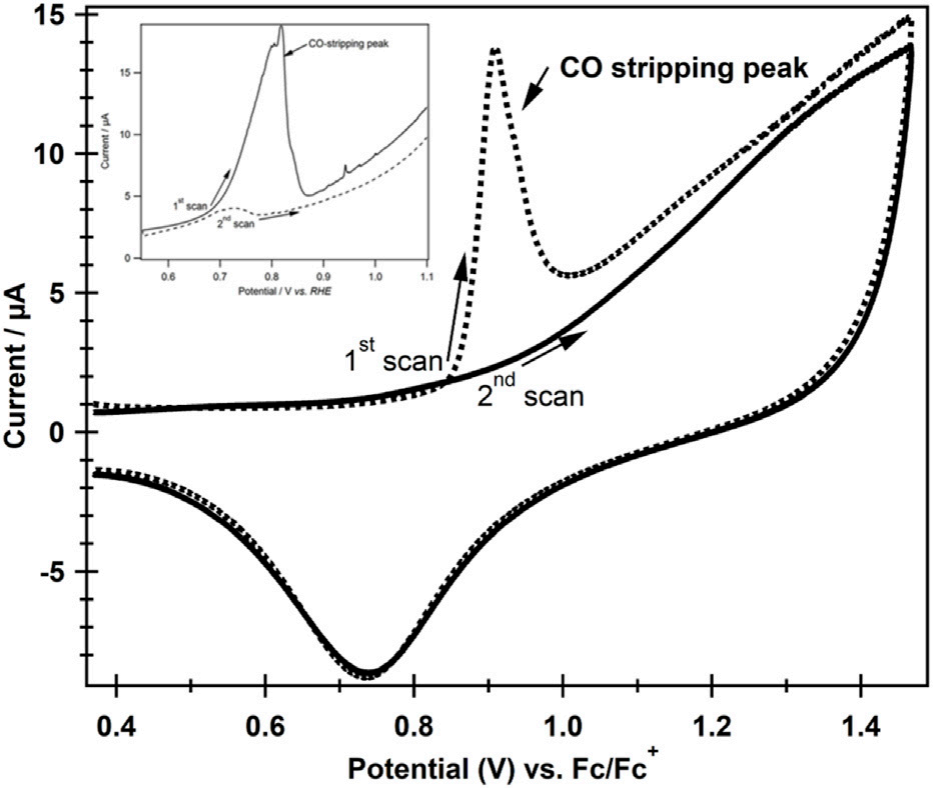
CVs on a Pt disk (having adsorbed CO) in a CO-free 0.1 M HClO_4_ (aq.) at a scan rate of 100 mV s^−1^ and 298 K. CO adsorption on Pt was previously established at −0.4 V vs. Fc/Fc^+^ during the electrochemical reduction of CO_2_ in [emim][EtSO_4_] at an Ag electrode (held at −2.33 V vs. Fc/Fc^+^ and 2,400 s). **Inset:** shows forward anodic sweeps for CO stripping in 0.5 M [emim][EtSO_4_] in AcN.

It is suggested by Rosen et al. (2012) that after a one-electron reduction in imidazolium-based ILs in acidic media, an intermediate complex with [emim]^+^ cation is formed, which further results in CO formation. In order to confirm this in our IL medium, we performed product analysis by using the cyclic voltammetry technique. Figure 4 shows cyclic voltammograms recorded at the Pt electrode that was held at −0.4 V vs. Fc/Fc^+^ during CO_2_ reduction at an Ag electrode in [emim][EtSO_4_] at 100 mV s^−1^. The figure shows that a strong oxidative wave started flowing during the first positive going scan (dotted line) at an onset potential of 0.846 V with a peak potential of 0.908 V vs. RHE. This sharp oxidative wave is attributed to the voltammetric oxidation of Pt-adsorbed CO. The adsorbed CO was completely stripped off the surface of Pt during the first scan, as no CO oxidation peak was observed in the second scan (solid line) in the same potential region, showing that the surface of Pt was CO-free during the second scan. It is well established that at potentials ≥0.85 V (here onset potential of CO oxidation), Pt surfaces oxidize to form PtOH_ads_/PtO_ads._ in aqueous acid solutions. The surface Pt hydroxide is reduced back to Pt on the reverse scan, as evident from the broad reductive wave in Figure 4, at an onset potential of approximately 1.0 V with a peak potential of 0.75 V. The oxygen-containing Pt surface acts as a catalyst to oxidize CO-covered Pt to CO_2_ during oxidative sweep by the Langmuir–Hinshelwood mechanism (Martindale and Compton, 2012; Samjeské et al., 2009). Formation of CO during CO_2_ERR in [emim][EtSO_4_] at the Ag electrode and its adsorption on and then removal from the Pt surface was reproducible, as a similar behavior was observed when the experiment was performed again at 323 K under the same set of other experimental conditions. In addition, we have shown that the CO_2_ reduction rate at the Ag electrode in [emim][EtSO_4_] increased as the overpotential gradually decreased with the increase in temperature of the system, probably due to a decrease in the viscosity of the liquid with rising temperature. The formation of CO was also detected during the electroreduction of CO_2_ at an Ag electrode in AcN containing 0.5 M [emim][EtSO_4_] as a supporting electrolyte (inset of Figure 4).

**FIGURE 5:**
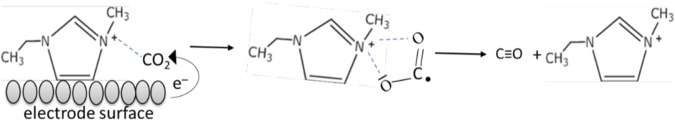
Mechanistic approach for CO_2_ ERR to CO in [emim][EtSO_4_] and CO_2_^·−^ stabilization interaction with [emim]^+^ cation.

Based on the proposed mechanism in the literature (Zhang et al., 2024), it is suggested that the CO_2_^−^ intermediate formed by a one-electron reduction in the [emim]^+^⋅⋅⋅CO_2_ medium at the Ag electrode is stabilized by forming an intermediates complex through interaction between CO_2_^·−^ and [emim]^+^. This can facilitate the binding of CO_2_^·−^ at the electrode interface to accept electrons for further reduction to CO. Additionally, during electrochemical reduction, the imidazolium cation can continue to exhibit catalytic activity because the structures of [emim]^+^ do not change. The schematics are shown in Figure 5. The optimization of CO_2_ conversion at the Ag electrode in terms of maximizing CO production would be beneficial to produce liquid fuels when combined with H_2_ via the Fischer–Tropsch process as well as by Sabatier’s process (Ma and Kenis, 2013).

## 4 Conclusion

In this work, it is shown that polycrystalline Ag and Cu are better catalysts for CO_2_ electroreduction in [emim][EtSO_4_] than Au, Pt, and BDD electrodes. By using a potential-step chronoamperometry, the diffusion coefficient (D) and concentration (C) of CO_2_ in [emim][EtSO_4_] were determined to be 4.78 m^2^ s^–1^ and 0.018 mol L^–1^, respectively. It is observed that Ag can reduce CO_2_ in AcN at a 200 mV lower potential in the presence of [emim][EtSO_4_] as a supporting electrolyte compared to a commercial supporting electrolyte, [TBA][PF_6_]. It is found that the CO_2_ reduction in AcN took place at a lower potential when we used imidazolium-based ionic liquids with [emim]^+^ cations than when using pyrrolidinium, an [empyrr]^+^-based ionic liquid. It can be concluded that the imidazolium-based ionic liquids, both as a solvent and as a supporting electrolyte in AcN, play a co-catalytic role in the reduction of CO_2_ at Ag electrodes at a lower overpotential. In addition, we confirmed that the cation of the ILs is responsible for lowering the overpotential during the CO_2_ERR by the ILs, while the role of the anions is not significant. Furthermore, it is found and verified electrochemically that CO is the product formed by the CO_2_ERR at an Ag electrode both in [emim][EtSO_4_] electrolyte and in the solution of [emim][EtSO_4_] in AcN. The product obtained can be reacted with green hydrogen to produce synthetic petrol by the Fischer–Tropsch process or methane by the Sabatier process (i.e., methanation of CO_2_) on a large scale in the future.

## Data Availability

The datasets presented in this study can be found in online repositories. The names of the repository/repositories and accession number(s) can be found in the article/Supplementary Material.
