# Supplementary material for: Efficient electrocatalytic reduction of CO2 on an Ag catalyst in 1-ethyl-3-methylimidazolium ethylsulfate, with its co-catalytic role as a supporting electrolyte during the reduction in an acetonitrile medium
Source: Front Chem. 2025 Apr 9;13:1515903. doi: 10.3389/fchem.2025.1515903 (PMC12015760; doi:10.3389/fchem.2025.1515903)
Supplement: Supplementary file 1 [file DataSheet1.docx]

**Supplementary Information**

**Efficient Electrocatalytic Reduction of CO₂ at Ag Catalyst in 1-Ethyl-3-Methylimidazolium Ethylsulfate and its Co-Catalytic Role as a Supporting Electrolyte in Acetonitrile Medium**

*Sayyar Muhammad*^1^* Asad Ali*^2^

*^1^Department of Chemistry, Islamia College Peshawar, 25120-Peshawar, Khyber-Pakhtunkhwa, Pakistan*

*^2^* Energy engineering, Division of Energy Science, Luleå University of Technology, 97187 Luleå Sweden

*** *Corresponding author: E-mail: sayyar@icp.edu.pk (S. Muhammad)*

*Email: asad.ali@associated.ltu.se (A. Ali)*

***Figure*** ***S1.*** *Cyclic voltammogram on an Ag electrode obtained at a sweep rate of 10 mV s^−1^ in a 5 mM Pb(NO_3_)_2_ solution in 10 mM KCl + 10 mM HNO_3_ in a potential range from 0.0 V to −0.75 V, showing the UPD and bulk deposition of Pb. Inset: shows only the Pb UPD region. The arrow shows the direction of the scan.*


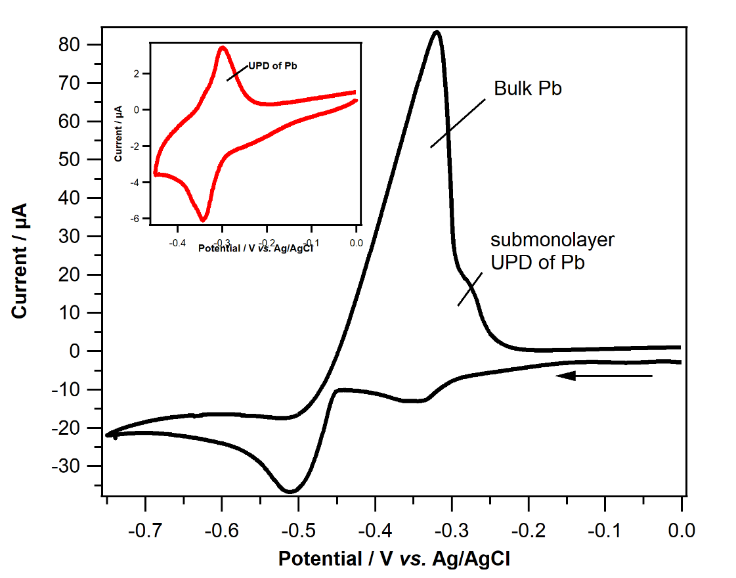


***Figure S2.*** *Cyclic voltammogram recorded at a 2 mm Cu electrode at 10 mV s^−1^ in a 5 mM Pb(NO_3_)_2_ solution in 10 mM KCl + 10 mM HNO_3_ in a potential range from, 0.0 V to −0.75 V, showing the UPD and bulk deposition of Pb. The arrow shows the direction of the scan.*


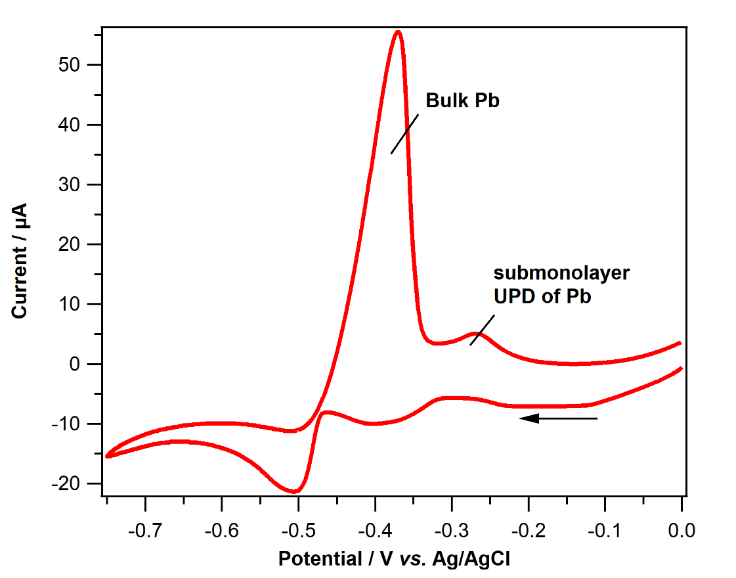


***Figure S3.*** *Cyclic voltammograms at a 2 mm diameter Pt (red line) and at a 2 mm diameter, Au (black line) disk electrodes at a scan rate of 100 mV s^−1^ and 25 °C in 5 mM ferrocene solution in [emim][EtSO_4_] in a potential range between 0.0 V and 0.5 V vs. Ag/Ag^+^. Inset: shows CVs adjusted vs. Fc/Fc^+^ redox couple.*


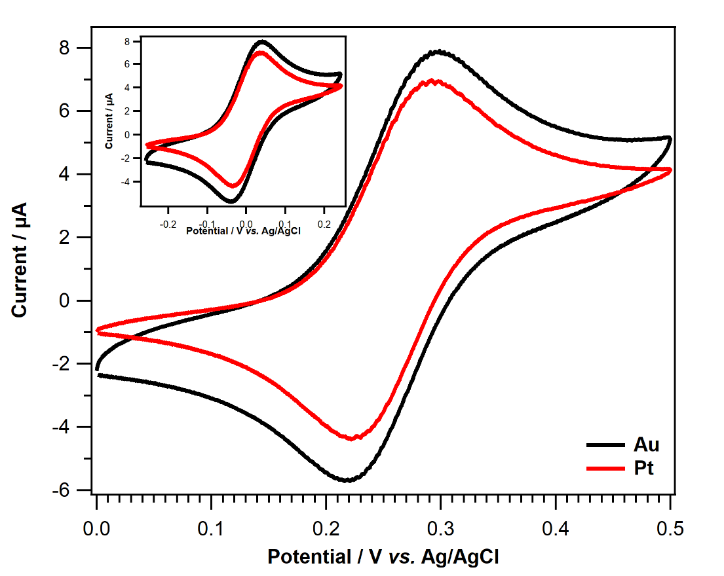


***Figure S4.*** *CVs obtained at 3 mm diameter GC electrode vs. Ag/Ag^+^ at a scan rate of 100 mV/s and 25 °C in a solution of (A) 0.1 M [emim][EtSO_4_] and (B) 0.1 M [TBA][PF_6_] in MeCN containing 5 mM ferrocene. The formal potential was 0.14 V in the case of (A) and (B) 0.11 V for Fc/Fc^+^ redox couple Inset: in each case shows CVs after adjusting the potential scale in each case vs. Fc/Fc^+^.*


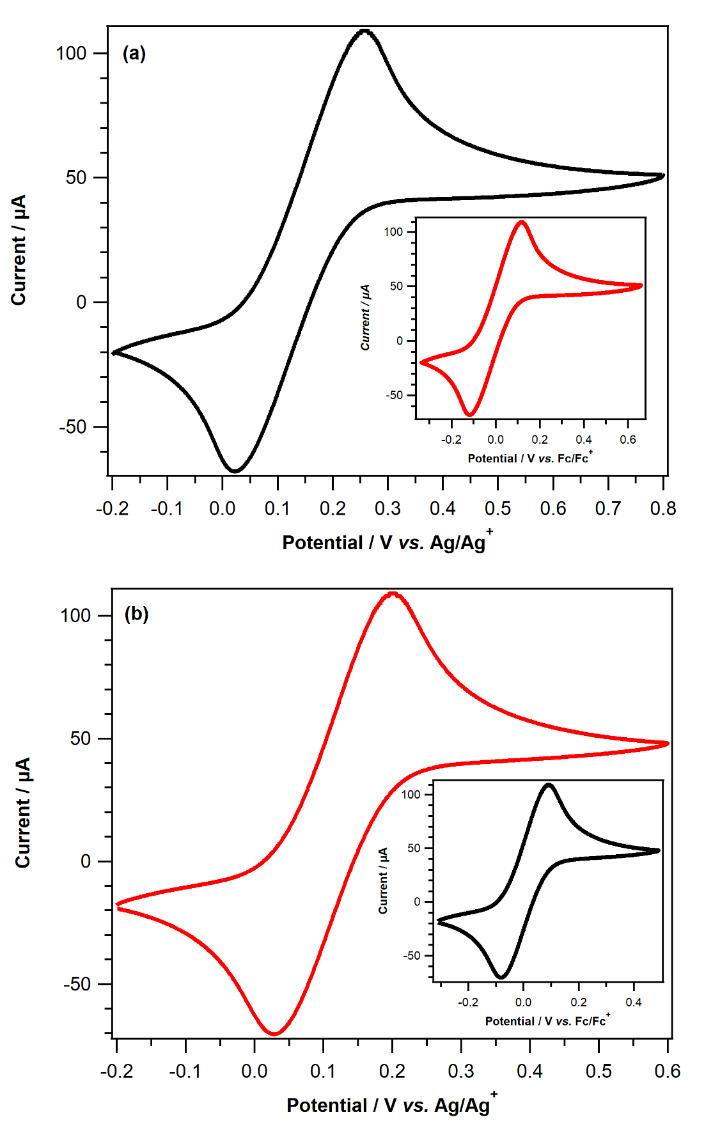


***Figure S5.*** *CV obtained in, 0.1 M KCl (aq.) containing 2 mM ferrocene at a 25 µm diameter Au electrode at a 5-mV s^−1^.*

**
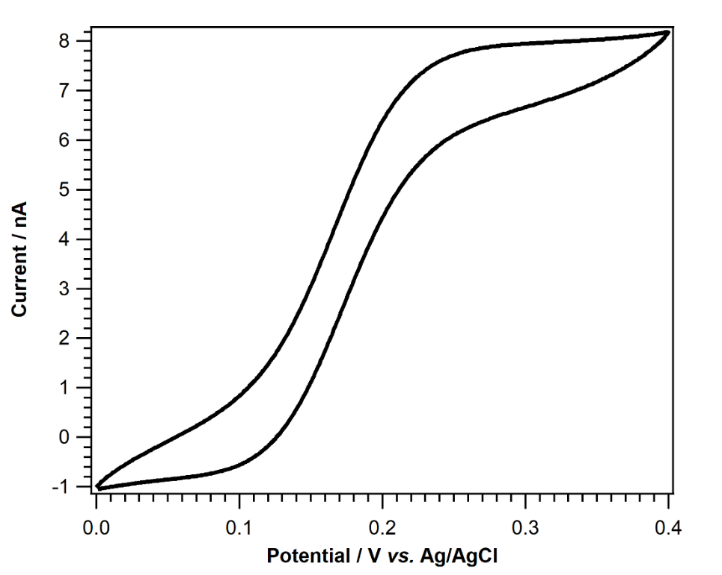
**
